# Successful intrathecal neurolytic block for the management of cancer pain in a 10-year-old child: a case report

**DOI:** 10.1186/s40981-021-00438-8

**Published:** 2021-04-12

**Authors:** Shogo Tashiro, Kohei Godai, Yukihisa Daitoku, Tomoyo Sato, Kei Enohata, Natsue Kiyonaga, Kenichi Maekawa, Yuichi Kanmura

**Affiliations:** 1 Department of Anesthesiology and Pain Medicine, Kagoshima University Hospital, 8-35-1 Sakuragaoka, Kagoshima, 890-8520 Japan; 2 Palliative Care Center, Kagoshima University Hospital, 8-35-1 Sakuragaoka, Kagoshima, 890-8520 Japan

**Keywords:** Intrathecal neurolytic block, Pediatric cancer pain, Pediatric palliative care

## Abstract

**Background:**

Cancer pain management in children is challenging owing to their unique patient characteristics. We present the case of a 10-year-old girl whose cancer pain was successfully managed using an intrathecal neurolytic block.

**Case presentation:**

The patient experienced severe cancer pain due to recurrent right ilium osteosarcoma. The tumor progressed rapidly despite chemoradiotherapy and gradually invaded the right lumbar plexus, which resulted in severe neuropathic pain in the right lower extremity. Systemic analgesics failed to attenuate the pain. We performed an intrathecal neurolytic block using 10% phenol-glycerol. The neurolytic block completely relieved her right lower extremity pain. After the block, the patient’s quality of life improved, and she spent her time with family.

**Conclusions:**

The intrathecal neurolytic block successfully relieved the patient’s cancer pain. Successful intrathecal neurolytic blocks require meticulous pain assessment of individual patients, to avoid possible serious complications such as paresis/paralysis and bladder/bowel dysfunction.

## Background

Cancer pain is one of the most intractable pains [1]. The mechanisms of cancer pain are complex and include mixed conditions of somatic, visceral, and neuropathic pain [2]. Meticulous pain assessment in individual patients is crucial for the successful management of cancer pain. Cancer pain management in children is especially challenging owing to their unique characteristics [3]. These characteristics include age, development, communication skills, and involvement of parents/caregivers. Opioids play a central role in the management of cancer pain. However, opioids are less effective in some pain conditions, such as neuropathic pain [4].

The intrathecal neurolytic block is an interventional procedure for the management of refractory cancer pain [5]. It involves the application of chemical agents in the subarachnoid space for degeneration of the targeted nerve fibers. Reports of the intrathecal neurolytic block in pediatric patients are limited [6, 7]. Here, we report a case of successful cancer pain management using an intrathecal neurolytic block in a 10-year-old child.

## Case presentation

**Fig. 1:**
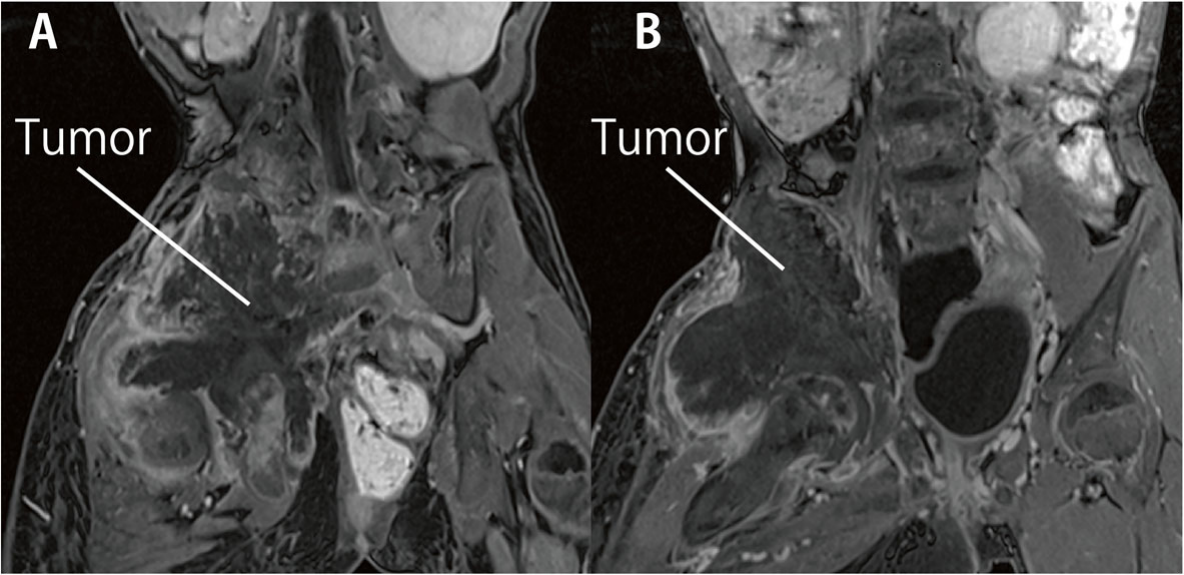
Magnetic resonance imaging of the tumor. Magnetic resonance imaging shows that the iliac tumor had invaded the right lumbar plexus. Slightly different sections are shown (**a** and **b**)

The patient was a 10-year-old girl (height, 118 cm; weight, 20 kg) with recurrent right ilium osteosarcoma, which spread to the right femur. She received chemoradiation therapy for right ilium osteosarcoma 3 years before presentation. The tumor gradually invaded the right lumbar plexus (Fig. 1). The spread of the tumor led to progressive lower extremity muscle weakness with throbbing pain in the right lower extremity. Metastasis of the tumor in both lungs was detected. The cancer was diagnosed as advanced, and end-of-life care was initiated. The attending pediatricians tried to manage the pain with acetaminophen, ibuprofen, pregabalin, ketamine, oxycodone, and fentanyl. The pain in the right lower extremity progressively worsened with time. The palliative care doctor gradually increased the dose of analgesics. The maximum dose of intravenous oxycodone was 1320 mg/day, which is equivalent to an oral morphine dose of 2640 mg/day. Benzodiazepines were used to mildly sedate the patient because pain control was poor. A urinary catheter was inserted due to urinary retention. The attending pediatricians estimated her life expectancy to be approximately 1–2 months. The patient and her parents expressed desire to be discharged home. However, due to intractable pain, the patient could not be discharged.

**Fig. 2:**
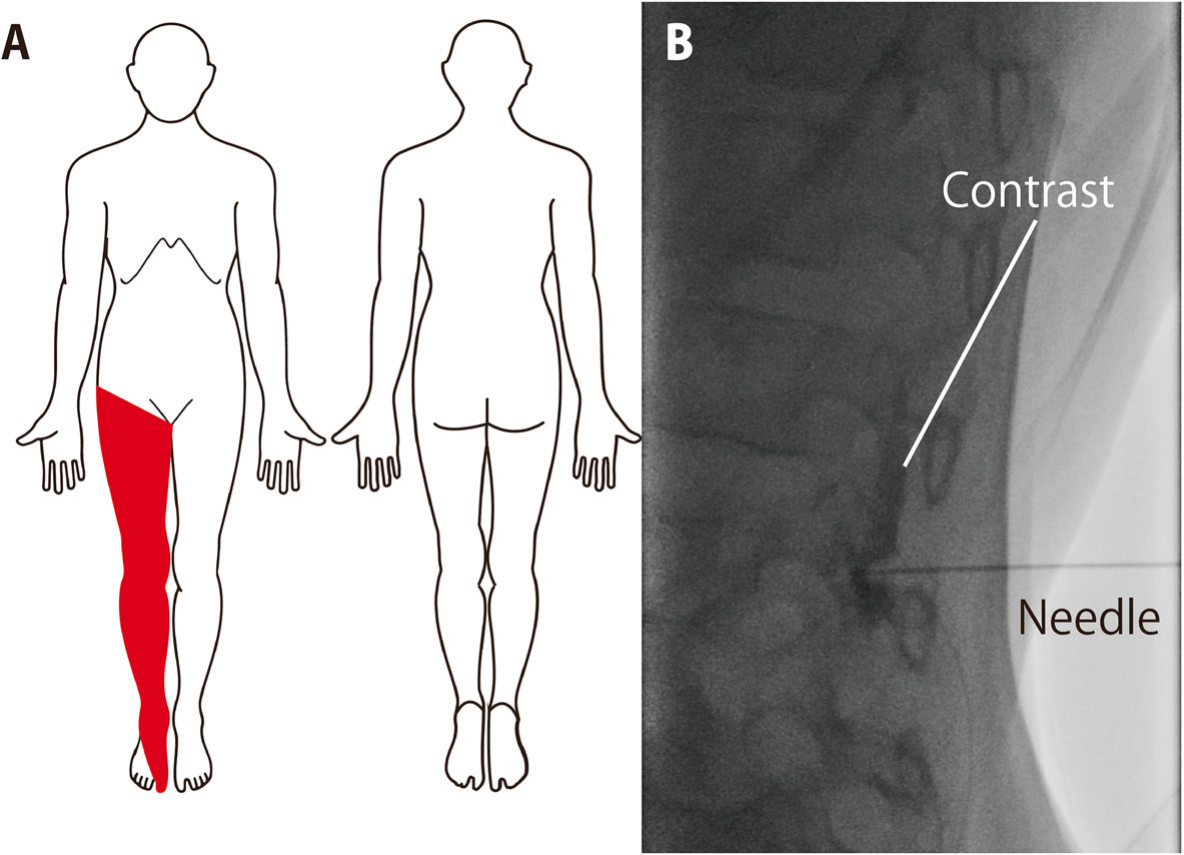
Pain area (**a**), contrast imaging during the test block (**b**). The patient’s pain area (**a**) and contrast imaging during the test block (**b**) are shown

She was then referred to the Department of Pain Medicine. Because the patient was shy and rarely communicated with the medical staff, we obtained information regarding her condition from her parents. The spread of the tumor led to progressive lower extremity muscle weakness (2 to 3/5 in the manual muscle test) with severe throbbing pain (NRS 7 to 9) (Fig. 2a). She was not able to walk because of muscle weakness. She also complained of general fatigue, malaise, and shooting pain (NRS 10). Shooting pain appeared more than five times per hour in the same region of throbbing pain. Mild allodynia was observed in the region of pain. A tactile sensation at the innervation territory of the right thoracic (T) 12 to lumbar (L) 5 spinal nerves was decreased to 5/10 compared to the contralateral side. We diagnosed her pain as a mixed condition of neuropathic pain and somatic pain. Severe shooting pain and mild allodynia were observed in the pain region. We classified her pain as “definite neuropathic pain” using a grading system for neuropathic pain published by The International Association for the Study of Pain [8]. The throbbing pain was considered to be somatic pain caused by the direct expansion of the tumor. She also experienced sleep disturbances due to the pain.

We considered regional anesthesia techniques to relieve the pain since systemic analgesic therapy failed to adequately attenuate it. There was no space to perform a peripheral nerve block because the tumor had spread near the spinal canal. We had three options: first epidural infusion, second intrathecal infusion, and third intrathecal neurolytic block. Epidural analgesia might not have been able to control the pain due to its relatively wide dermatomal spread. Catheter management may have been troublesome. Although neurolytic block may cause paresis/paralysis and bladder/bowel dysfunction, she was not able to walk due to muscle weakness. She already had a urinary catheter and constipation. We considered that paresis/paralysis and bladder/bowel dysfunction were acceptable complications. After discussing the benefits and risks with the patient, parents, palliative care doctors, and attending pediatricians, we decided to try an intrathecal neurolytic block.

To evaluate the efficacy of intrathecal analgesia, we performed a test block using a fluorescence-guided intrathecal nerve block. On arrival in the procedure room, pulse oximetry, electrocardiography, and noninvasive blood pressure monitoring were established. The patient’s mother accompanied her and stayed in the room during the procedure. After applying oxygen at 7 L/min using a facemask, moderate sedation was initiated with intravenous thiamylal (75 mg). Moderate sedation with spontaneous ventilation was maintained using intermittent thiamylal (total dose, 200 mg). We selected thiamylal as a sedative because the patient already received thiamylal several times. We carefully monitored patient’s airway and ventilation. We changed the patient’s position from the supine position to the right lateral decubitus position. After local anesthesia with 1% mepivacaine, a 23 G Quincke needle was inserted into the L 3/4 intervertebral space. We confirmed adequate spread (L 2 to L 4 levels) of contrast using 24% iotrolan (0.3 mL) (Fig. 2b). Hyperbaric bupivacaine (0.5%, 0.3 mL) was injected intrathecally. We kept her in the right lateral decubitus position for an hour. Her vital signs were within the normal range during the procedure. The patient did not complain of any pain for 6 h. The intrathecal nerve block was considered to be effective.

Five days after the test block, we performed the intrathecal neurolytic block. The patient was mildly sedated using intravenous midazolam (2 mg) because the patient seemed anxious. Oxygen 3 L/min was applied via a facemask. Moderate sedation was maintained with intermittent thiamylal administration (total dose, 300 mg). Airway obstruction or insufficient ventilation was not observed. After local anesthesia with 1% mepivacaine, a 22 G Quincke needle was inserted into the L 3/4 intervertebral space. A 0.2 mL solution of 10% phenol-glycerol was injected. We maintained her in the right lateral decubitus position for 2 h. Her vital signs were within the normal range during the procedure. The patient reported decreased touch and pain sensation from T 12 to L 5 in the dermatome without any signs of motor block.

Over the following few days, intravenous administration of oxycodone was significantly reduced. The neurolytic block completely relieved her right lower extremity pain. Finally, she received only transdermal fentanyl patch 2 mg/day, which is equivalent to an oral morphine dose of 60 mg/day due to low back pain. The complication of neurolytic block was not observed. The patient was discharged 2 weeks after the neurolytic block. She was able to go outside using a wheelchair. Her quality of life improved and spent her last time with family. She died 3 months after the neurolytic block was performed.

## Discussion

We present a pediatric cancer patient whose cancer pain was successfully managed with an intrathecal neurolytic block. The patient’s cancer pain included neuropathic pain and nociceptive somatic pain. Systemic analgesic therapy failed to adequately attenuate the pain. However, an intrathecal neurolytic block using phenol completely relieved the right lower extremity pain. After the neurolytic block, the patient’s quality of life improved, and she spent her last time with family without severe pain.

This case report highlights that clinicians need to assess pediatric cancer pain with caution. We discussed about possible treatment options in detail with parents, the patient, attending pediatricians, and palliative care doctors. We carefully let parents and the patient take their time to make a decision. Although indication for the intrathecal neurolytic blocks for children is not different from that for adults, meticulous assessment and discussion are needed for children.

Intrathecal neurolytic block interrupts the input of pain from injured tissues at the spinal cord [5]. Some researchers claim that intrathecal neurolytic block is less effective in neuropathic pain than somatic pain [9]. The study, however, was conducted in the 1970’s, several decades before the guidelines of neuropathic pain was established [8]. Recent case report shows that intrathecal neurolytic block is effective in neuropathic cancer pain [10]. Intrathecal neurolytic block completely relieved neuropathic and somatic pain in the patient. There are serious complications associated with intrathecal neurolytic block. These complications include paresis/paralysis, bladder dysfunction, and bowel disorders [5]. Intrathecal neurolytic block was indicated in our patient because she already had paresis of the right leg and had a urinary catheter. We did not select sympathetic nerve blocks in the patient. It is possible that the patient’s pain includes sympathetically mediated pain. However, the main component of the patient’s pain was somatic and neuropathic pain. We considered that it was difficult to control the pain using a sympathetic nerve block.

## Conclusions

In conclusion, intrathecal neurolytic block successfully relieved the patient’s cancer pain. After the neurolytic block, the patient’s quality of life improved, and she spent her time with family without severe pain. Successful intrathecal neurolytic blocks require meticulous pain assessment of patients, to avoid possible serious complications such as paresis/paralysis and bladder/bowel dysfunction.

## Data Availability

Not applicable.

## Abbreviations

L: Lumbar
NRS: Numerical rating scale
PCA: Patient-controlled analgesia
T: Thoracic
